# Supplementary material for: Safflower Flavonoid 3′5′Hydroxylase Promotes Methyl Jasmonate-Induced Anthocyanin Accumulation in Transgenic Plants
Source: Molecules. 2023 Apr 4;28(7):3205. doi: 10.3390/molecules28073205 (PMC10095914; doi:10.3390/molecules28073205)
Supplement: Supplementary file 1 [file molecules-28-03205-s001.zip › molecules-2275049-supplementary.pdf]

Supplementary Figures and Tables

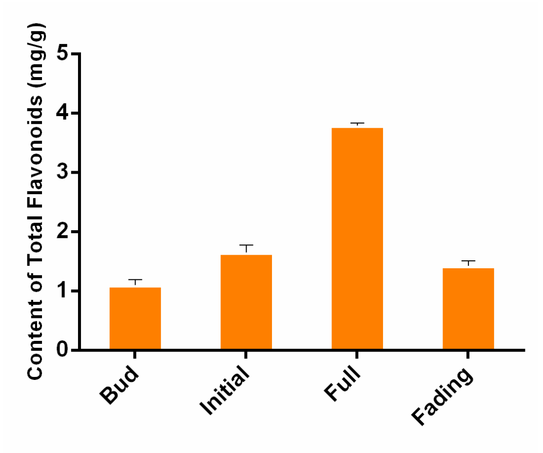

Figure S1. The total flavonoids content in different flowering periods (bud, initial, full and fading).

```
1      MTPNFGDKIY ISMINLWSWW
21     WQADNHQDHV ARTILTVSVP
41     LLVFLWYQLT ELYIKKGRTP
61     LPPGPYGLPV VGYLPFLSSD
81     VHERFTHMSH RYGPIFSLWL
101    GSKLHVVVNS MDLARVVARD
121    RDQTFANRNP PITALTITSG
141    APDVVWSSNN AHWRGMRKLL
161    VSQVLSNANL NSCAGFRTDA
181    VRKAVREVYG RIGERIDINK
201    VAFDAELNVV TGMLWGCCDW
221    KGSSDVIGEG FREVEFKIIE
241    LMGAPNVSDF FPMLSWFDLQ
261    GREREMRKQT EHLHRIVDKI
281    IGGRSNGNFR KIGEDERKDF
301    VQILLELKEQ KDGSISIEQI
321    KGLLFDILIA TTDTTSTMAE
341    WVMTEILHHP DVKTKIQEEL
361    NDVLGMNNIV EECHLGKLTY
381    LDAVIKETFR IHSPLPLLIP
401    RCPDEPCTVG GYLIPKGTIV
421    YINVWAIHRD PKNWSEPLKF
441    KPERFLNGKW DYSGNNLKFL
461    PFGVGRRICP GIQLGEKMLV
481    YILASLLHSF EWGLPKGEDF
501    EVSDEFGFVT KKRKPLIAIP
521    SQRLSNTNLY L*
```

Figure S2. Amino-acid sequence of the CtF3’5’H1protein.

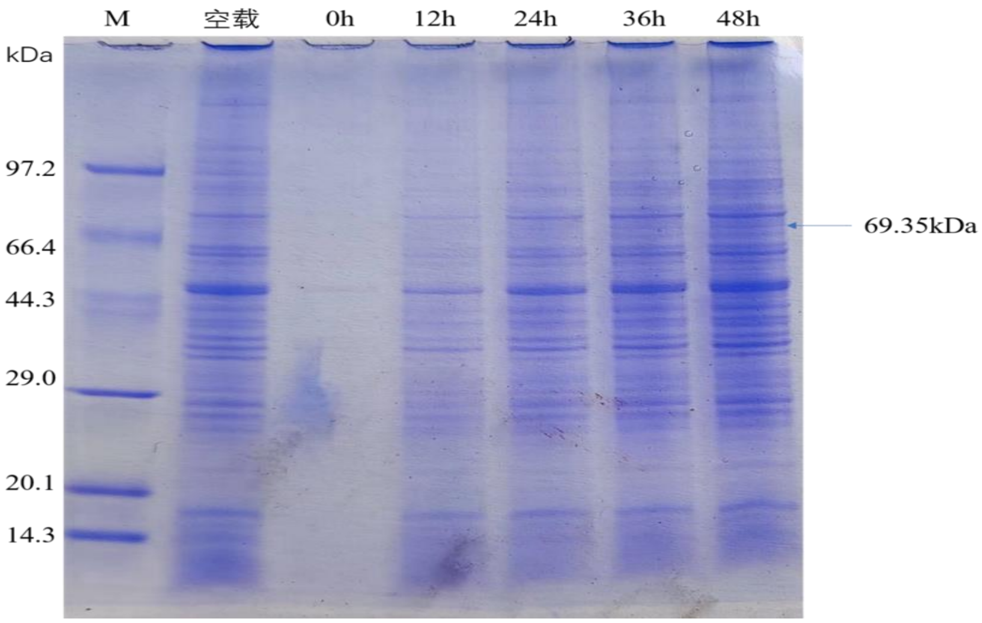

Figure S3. SDS-PAGE image of recombinant CtF3’5’H1. First line is Protein marker; the second is the empty vector; 0 hour, 24 hours, 36 hours, 48hours.

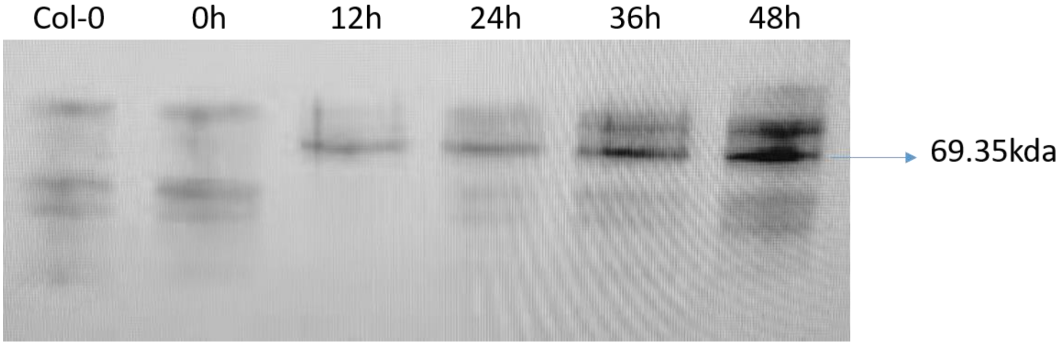

Figure S4. Part of Western Blot validation diagram. Figure S2. First line is the empty vector; the second is 0 hours, next to 12h, 24h, 36h, 48h.
There are specific bands at 69.35kDa.

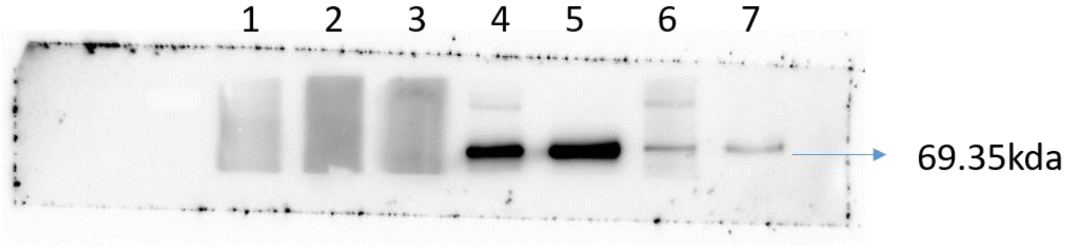

Figure S5. Detection after purification. 1: protein marker (10~170kDa). 2: col-0(the empty vector). 3: 0h. 4,6: binding buffer. 5,7: elution buffer

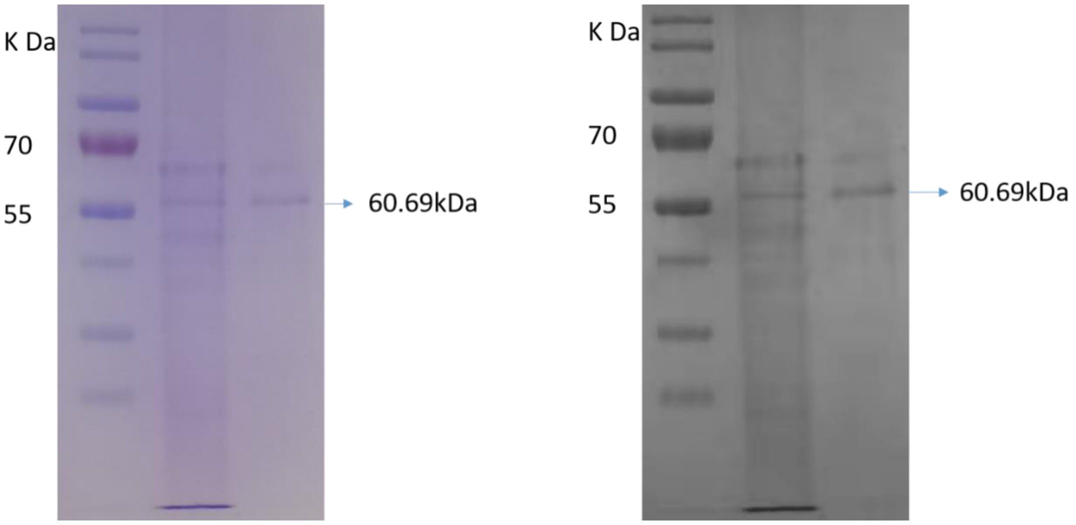

Figure S6. Purification by SDS. The size of the purified protein is the same as that after the removal of two his tags. There is only the clearest one, and the size is 60.69 kDa.

a

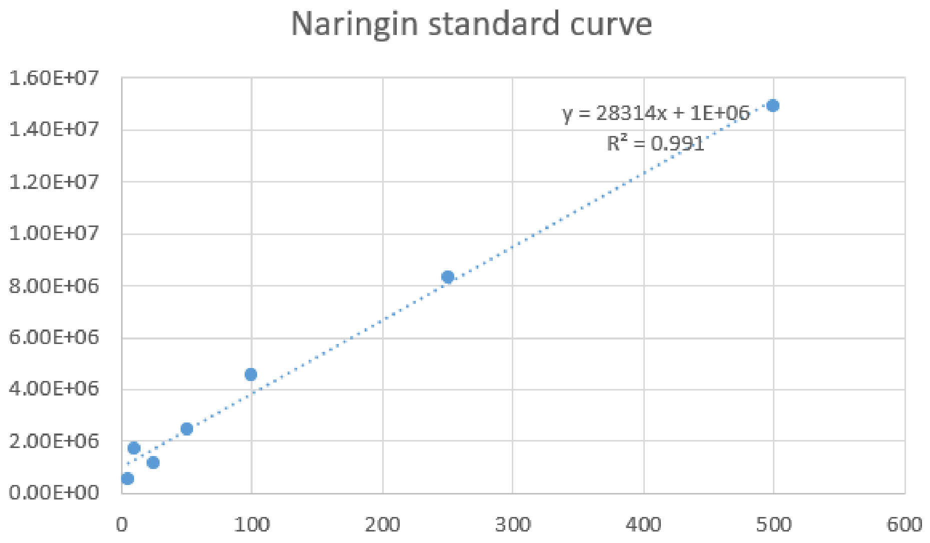

b

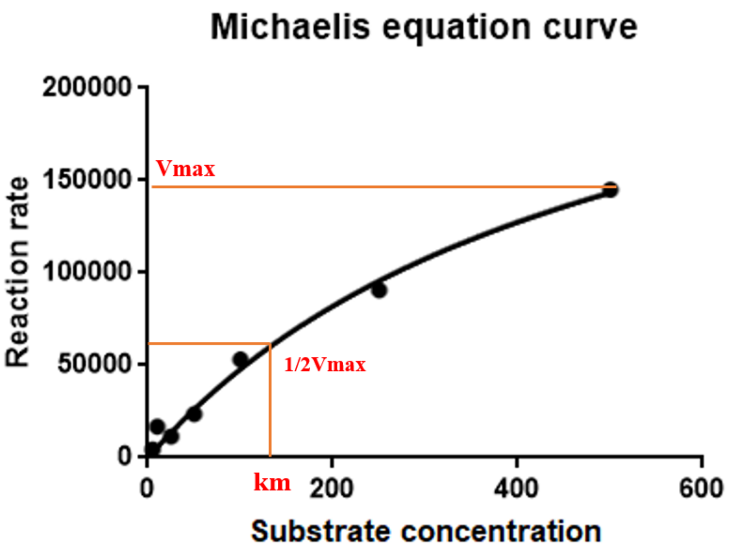

Figure S7. The Naringin standard curve and Enzyme kinetic curve.

As shown in the figure a, the standard curve of the peak area produced by the external standard method for different concentrations of substrates is shown. Abscissa represents substrate the concentration, ordinate represents peak area. (R^2= 0.991.) As shown in the figure b, the Abscissa is the substrate concentration, the ordinate is the reaction rate, and the maximum reaction rate and the substrate concentration when the maximum reaction rate is half of the maximum reaction rate have been marked.

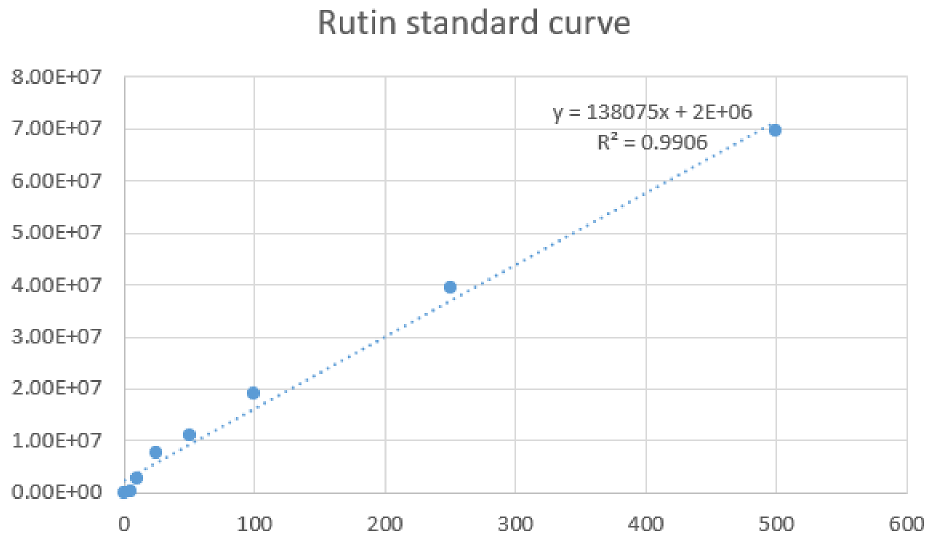

Figure S8. The RUTIN standard curve. The standard curve of total flavone plotted by seven Rutin standard substances with different concentrations (5ng/ml, 10ng/ml, 25ng/ml, 50ng/ml, 100ng/ml, 250ng/ml, 500ng/ml).

Table S1 List of primers used in this study.

Gene	Sequence (5'-3')
18srRNA-F	GAGAAACGGCTACCACATCCAA
18srRNA-R	TCGTTTGAGCCCGGTATTGTTA
CtF3'5'H1-F	AGATGAACCTTGCACAGTTGGC
CtF3'5'H1-R	ACGGCTCAGACCAGTTTTTGG
CtF3'5'H2-F	CGTCGCTAGCAATGCAAGTTG
CtF3'5'H2-R	ATGGCTACTACAATCCCGGCA
CtF3'5'H3-F	AAGATCTGGCTCGGAAGCAAG
CtF3'5'H3-R	AGGTTGCGATTCGCAAAGG
CtF3'5'H4-F	ATGGTGGTGGCAAGTGATGAA
CtF3'5'H4-R	AGCGGTACCATAAAACGACCG
CtF3'5'H5-F	CGATTGTTGGCTACCTTCCGT
CtF3'5'H5-R	GCTTCCGAGCCAGATCTTGAA
CtF3'5'H6-F	ACCTTCGCCAACCGTAATCCT
CtF3'5'H6-R	TTACGCATGTTACGCCAGTGC
CtF3'5'H7-F	TGGTCCGAATAACATCGTCGA
CtF3'5'H7-R	GGAGAGGAACCGGAGGTTGTAA
CtF3'5'H8-F	ACCTTCCGTTTCTTAGCCCTGA
CtF3'5'H8-R	GGCTGCTTATCACGACGAAGAG
CtF3'5'H9-F	AACATTTCGGTTGCACCCC
CtF3'5'H9-R	AAAGACGGTACAGCCCTTTGG
CtF3'5'H10-F	CCATTTCTAGGCCCTAACCTGC
CtF3'5'H10-R	ACCACGATGTGGAGCTTACTGC
CtF3'5'H11-F	GATCAAACTCAACGAGGCCGT
CtF3'5'H11-R	GCAGCTCAGCCACATGATCAT
CtF3'5'H12-F	TGTGGCTGAGTTGCAGACGAT
CtF3'5'H12-R	TCCCCTGAAGATCCAACCATG
CtF3'5'H13-F	GGTCGCTGAACGTTATGACGA
CtF3'5'H13-R	AAGAAGCTCCACGATCCTCGA
